# Genetic Diversity of *Staphylococcus aureus* Strains from a Tertiary Care Hospital in Rawalpindi, Pakistan

**DOI:** 10.3390/microorganisms9112301

**Published:** 2021-11-05

**Authors:** Muhammad Ali Syed, Bushra Jamil, Hazem Ramadan, Maria Rukan, Shahzad Ali, Shahid Ahmad Abbasi, Tiffanie A. Woodley, Charlene R. Jackson

**Affiliations:** 1 Department of Microbiology, The University of Haripur, Haripur 22620, Pakistan; mirwah2000@yahoo.de (M.A.S.); mahirazaman404@gmail.com (M.R.); 2 BJ Micro Lab (SMC Pvt) Limited, Islamabad 44000, Pakistan; Bushrajamil2000@gmail.com (B.J.); shahidahmadabbasi@yahoo.com (S.A.A.); 3 Hygiene and Zoonoses Department, Faculty of Veterinary Medicine, Mansoura University, Mansoura 35516, Egypt; hazem_hassan@mans.edu.eg; 4 Bacterial Epidemiology and Antimicrobial Resistance Research Unit, USDA-ARS, US National Poultry Research Center, Athens, GA 30605, USA; tiffanie.woodley@usda.gov; 5 One Health Research Group, Discipline of Zoology, Department of Wildlife and Ecology, University of Veterinary and Animal Sciences, Lahore 54000, Pakistan; shahzad.ali@uvas.edu.pk

**Keywords:** *Staphylococcus aureus*, antibiotic resistance, multilocus sequence typing, pulsed-field gel electrophoresis

## Abstract

*Staphylococcus aureus* is an important healthcare-associated bacterium that causes a multitude of infections in humans such as superficial skin and soft tissue infections, necrotizing pneumonia, foodborne illnesses and postsurgical infections. Treatment of *S. aureus* infections has become more complicated due to the emergence of Methicillin-Resistant *Staphylococcus aureus* (MRSA), some of which are multidrug resistant. The present study aimed to characterize *S. aureus* isolates from a tertiary care hospital in the Rawalpindi district of Pakistan. Staphylococci were isolated from 300 clinical samples collected from January 2018 to January 2019 and *S. aureus* isolates were tested for antimicrobial susceptibility and analyzed using Pulsed-Field Gel Electrophoresis (PFGE), Multi-Locus Sequence Typing (MLST), staphylococcal cassette chromosome *mec* (SCC*mec*) and *spa* typing. Approximately 25.3% (76/300) of the clinical samples were positive for *S. aureus*; of those, 88.2% (67/76) were *mec*A+ (MRSA). In addition to the β-lactam antibiotics, high levels of resistance were also found to the fluoroquinolones (ciprofloxacin, gatifloxacin and levofloxacin (73.7% each)). Of the 23 different *spa* types identified, the majority of isolates belonged to *spa* type t632 and t657 (9/66; 13.6% each *spa* type). ST772-t657 (Bengal Bay clone) was the most commonly identified clone in this study although other clones circulating around different regions of the world were also found indicating the diversity in MRSA isolates from this area of Pakistan. This study emphasizes the need to monitor MRSA in the clinical setting for improved infection control and treatment options.

## 1. Introduction

*Staphylococcus aureus* is a well-known commensal found on different parts of the human body such as skin, skin glands, nose and gut mucus membranes [1]. However, *S. aureus* is a major cause of skin diseases and invasive infections, such as endocarditis, pneumonia and osteomyelitis, in both healthcare and community settings. It has been estimated that 20% of humans are persistent nasal carriers for *S. aureus,* while 30% are intermittent and 50% are non-carriers for *S. aureus* [2]. Persistent and intermittent colonization increases the opportunity for infection, which is primarily caused by the same strains present as a commensal on an individuals’ body [3,4,5]. Different infections, skin lesions and injuries, catheters, implants and chronic diseases (diabetes, AIDS, innate and acquired deficiencies of immune system) favor *S. aureus* infections [6,7].

Emergence of resistant phenotypes is usually linked with injudicious use of antimicrobial agents. A study by Schentag et al. (1998) reported changes in normal biota of a patient within 24–48 h under antibiotic pressure [8]. Some studies have supported causal relationships between antibiotic administration and emergence of Methicillin-Resistant *S. aureus* (MRSA) [9,10]. MRSA emergence was first observed in 1961, soon after the clinical application of penicillinase-resistant penicillin [1]. Patients infected with these resistant bacteria take longer to recover as compared to those infected with other staphylococcal species, especially those that are susceptible to antibiotics [9]. As such, MRSA isolates have been recognized as a source of infections with resistance to antibiotics in the β-lactam antibiotic class as a main characteristic contributing to its disease-causing ability along with other virulence factors in the bacterium [9].

The presence of mobile genetic elements also plays a major role in conferring resistance to antibiotics in MRSA such as resistance to vancomycin [7]. Mobile genetic elements include plasmids, transposons, bacteriophage and pathogenicity islands [5]. MRSA isolates also contain a mobile genetic element, Staphylococcal cassette chromosome (SCC*mec*), that may be horizontally disseminated among *S. aureus* isolates resulting in spread of antimicrobial resistance genes among the isolates [11]. SCC*mec* is composed of two parts, namely the *mec* gene complex and cassette chromosome recombinase (*ccr*) gene complex both of which contribute to production of different variants of MRSA.

Studies conducted in Pakistan in the last decade have reported high prevalence of MRSA [12,13,14,15,16]. Recent studies in our laboratory conducted on MRSA isolates from Peshawar and Malakand cities using microarray technology showed epidemiological links to the Middle Eastern/Arabian Gulf region [17,18]. However, available data for this region is still limited and there is a need for continued surveillance of *S. aureus* and characterization of isolates from local hospitals for control and better treatment options. The aim of the present study was to characterize clinical *S. aureus* isolates from a tertiary care hospital in Rawalpindi city of Pakistan. *S. aureus* from clinical samples were isolated and analyzed using antimicrobial susceptibility testing, presence of antimicrobial resistance genes, Pulsed-field Gel Electrophoresis (PFGE), Multi-Locus Sequence Typing (MLST), SCC*mec* and *spa* typing.

## 2. Materials and Methods

### 2.1. Sample Collection and Bacterial Identification

Three hundred clinical samples (urine, pus, tracheal tubes, vaginal swabs, body fluids, blood and cannula) were collected from January 2018 to January 2019 from Fauji Foundation hospital, Rawalpindi, Punjab, Pakistan. Samples were collected from patients for routine procedures to more urgent conditions including infected wounds, abscesses, burns and serious or life-threatening medical conditions. The samples were streaked onto Mannitol Salt Agar (MSA) using sterile cotton swabs and incubated at 37 °C for 24 h. For liquid samples (such as urine, blood, etc.), 50 μL of the sample was placed onto the surface of the MSA plate and then spread on the media surface using a sterile swab. Initial bacterial identification was performed by microscopy and biochemical tests (catalase test, tube coagulase test, mannitol fermentation and DNase). Confirmation of the isolates was performed by PCR using previously reported species-specific primers for *S. aureus* [19]. Pure cultures were preserved in glycerol stock at −80 °C. The work was approved by the Ethics Review Committee of Department of Microbiology of The University of Haripur.

### 2.2. Antibiotic Susceptibility Testing

Minimum inhibitory concentration (MIC, µg/mL) for *S. aureus* was determined by broth microdilution with the Sensititre^TM^ semi-automated antimicrobial susceptibility system (Trek Diagnostic Systems, Inc., Cleveland, OH, USA) and the Sensititre^TM^ Gram-Positive Plate GPN3F. Antimicrobials and breakpoints were: ampicillin (≥5 µg/mL), ceftriaxone (≥64 µg/mL), ciprofloxacin (≥4 µg/mL), clindamycin (≥4 µg/mL), daptomycin (>1 µg/mL), erythromycin (≥8 µg/mL), gatifloxacin (≥2 µg/mL), gentamicin (≥16 µg/mL), levofloxacin (≥4 µg/mL), linezolid (≥8 µg/mL), oxacillin (≥4 µg/mL), penicillin G (≥0.25 µg/mL), rifampin (≥4 µg/mL), streptomycin (≥1000 µg/mL), Synercid (Quinupristin/Dalfopristin (Q/D)) (≥4 µg/mL), tetracycline (≥16 µg/mL), trimethoprim/sulfamethoxazole (≥4/76 µg/mL) and vancomycin (≥16 µg/mL). MIC values were manually recorded using the Sensitouch system. Clinical and Laboratory Standards Institute (CLSI) standards were used to determine resistance [20,21]. Only susceptible breakpoints for daptomycin (<1 µg/mL) have been established by CLSI; resistance for this drug was defined as MICs greater than that value. *S. aureus* ATCC 29213 was used as a quality control strain [20,21].

### 2.3. Molecular Characterization

Multiplex PCR was used to test for the presence of resistance genes to aminoglycosides (*aacA-aphD*), macrolides (*erm*(A), *erm*(C))*,* oxacillin (*mec*A)*,* streptogramins (*vat*(A), *vat*(B), *vat*(C)) and tetracycline (*tet*(K), *tet*(M)) [22]. PFGE was used to generate macro-restriction patterns using 30 U of *Sma*I (Roche, Indianapolis, IN, USA) as previously described [23]. Cluster analysis was performed with BioNumerics software v6 (Applied Maths, Sint-Martens-Latem, Belgium) using Dice coefficient and the unweighted pair group method (UPGMA). Optimization settings for dendrograms were 2% with a band tolerance of 2%. SCC*mec* type [24], *spa* type [25,26], MLST/clonal complexes (CC) [27] were performed as previously described.

## 3. Results

### 3.1. Bacterial Isolation and Identification

Of the 300 samples collected, 76 (25.3%) were positive for *S. aureus*. Although the number of samples per source varied, the majority of positive samples were from pus (41.1%; 51/121), tracheal tubes (19.4%; 6/31), vaginal swabs (18.2; 2/11) and urine (16.7%; 1/6). Samples from blood (14.3%; 5/35), body fluids (12.7%; 7/55) and cannula (9.8%; 4/41) were also positive for *S. aureus*.

### 3.2. Antimicrobial Susceptibility Testing

**Figure 1:**
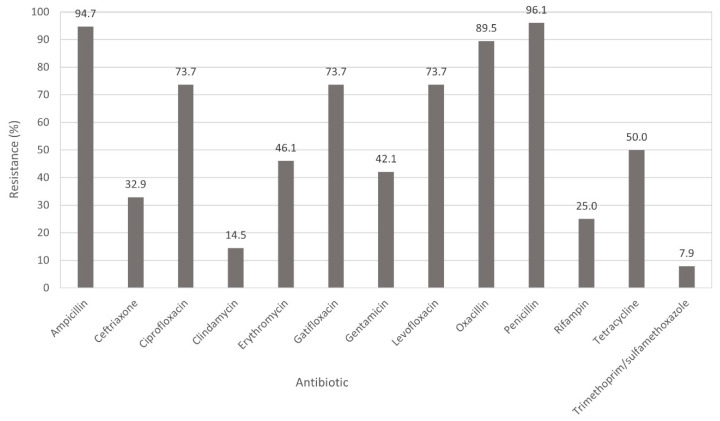
Percent resistance of *Staphylococcus aureus* (*n* = 76) to tested antimicrobials using broth microdilution. No resistance to daptomycin, linezolid, high-level streptomycin, Quinupristin/Dalfopristin, tetracycline, or vancomycin was detected.

Percent resistance of *S. aureus* isolates (*n* = 76) to the tested antibiotics is shown in Figure 1. Higher frequencies of antibiotic resistance were observed to ampicillin (94.7%; 72/76), oxacillin (89.5%; 68/76), ciprofloxacin (73.7%; 56/76), gatifloxacin (73.7%; 56/76) and levofloxacin (73.7%; 56/76). Moderate frequencies of antibiotic resistance ranging from 30% to 60% were found against tetracycline (50%), erythromycin (46.1%), gentamicin (42.1%) and ceftriaxone (32.9%). The lowest resistance observed was identified to rifampin (19; 25%), clindamycin (11; 14.5%) and trimethoprim/sulfamethoxazole (6; 7.9%). All isolates were susceptible to daptomycin, linezolid, high-level streptomycin, Q/D and vancomycin. Multidrug resistance (MDR; resistance to two or more antimicrobial classes) was observed with most common combinations of aminoglycoside/β-lactams/fluoroquinolones/macrolide-lincosamide-streptogramin/rifampin/tetracycline (Table 1). MDR patterns AmpCefCipCliEryGatGenLevOxaPenRifTet and AmpCefCipEryGatGenOxaPenRifTet contained eight MRSA isolates and one methicillin-susceptible *S. aureus* (MSSA) isolate for each pattern. One MRSA isolate was resistant to seven antibiotic classes (AmpCefCipCliEryGatGenLevOxaPenRifTetTri).

### 3.3. Frequency of Antibiotic Resistance Genes

Of the *S. aureus* isolates, 88.2% (67/76) were *mec*A+ (MRSA), while nine (11.8%) were negative for *mec*A and classified as MSSA. Isolates were also positive for *aacA-aphD* (34.2%), *erm*(A) (22.3%)*, erm*(C) (15.8%), *tet*(K) (13.2%) and *tet*(M) (23.7%). No PCR positive results were found for the streptogramin resistance genes, *vat*(A)*, vat*(B) and *vat*(C).

### 3.4. Molecular Analysis

**Figure 2:**
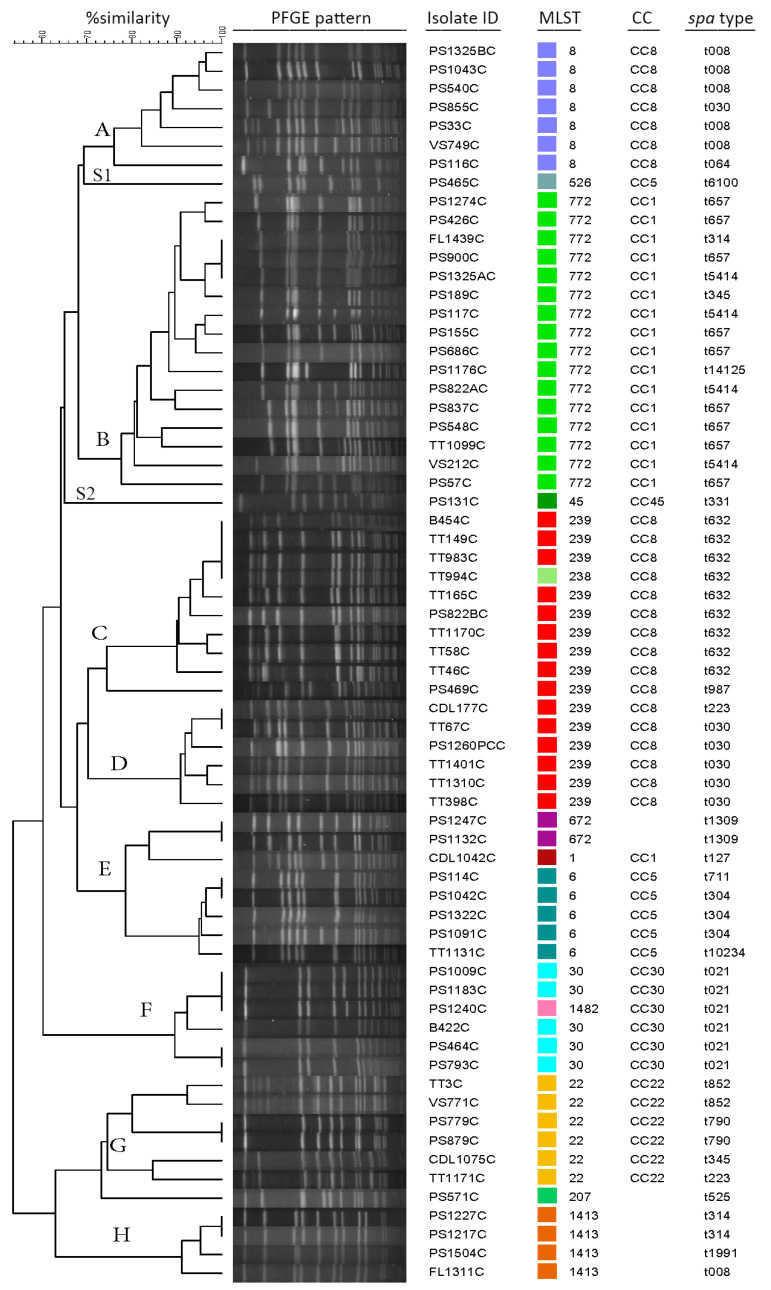
Pulsed-field Gel Electrophoresis (PFGE) analysis and genetic profiles (Multilocus Sequence Typing (MLST), clonal complex (CC), and *spa* types) of MRSA from clinical samples. DNA for PFGE was digested with *Sma*I. Levels of similarity were determined using Dice coefficient and the unweighted pair group method (UPGMA). PFGE clusters were determined using a 75% similarity cutoff and labeled A–H. Sample source Isolate ID: B = blood, CDL = cannula double lumen, FL = fluid. TT = tracheal tube, VS = vaginal swab.

**Figure 3:**
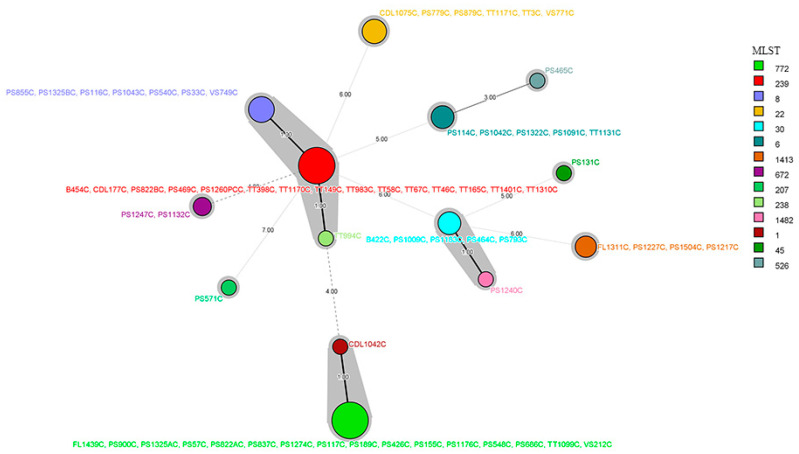
Minimum spanning tree (MST) of Multilocus Sequence Typing (MLST) of the examined clinical *Staphylococcus aureus* from humans. Each circle corresponds to an individual MLST type; the circle size is proportional to the number of isolates belonging to the same MLST type. The color of each circle denotes the MLST type. Connecting lines (solid and dashed) between circles represents allelic variations between MLST types; the grey shadowing indicates no more than one different loci between MLST types.

PFGE was conducted on all but one MRSA isolate as the isolate did not produce a useable PFGE pattern. Results showed the clustering of MRSA isolates (*n* = 66) based on 75% similarity into eight clusters (A, B, C, D, E, F, G, H) and two singletons (S1, isolate PS465C and S2, isolate PS131C) (Figure 2). Two PFGE clusters encompassed approximately 40% (26/66) of the examined isolates; clusters B and C contained 16 and 10 isolates, respectively. Other isolates were distributed into six clusters as follows: eight isolates in cluster E, seven isolates each in clusters A and G, six isolates each in clusters D and F and four isolates in cluster H. *spa* types among the MRSA isolates were highly diverse as isolates were distributed among 23 different *spa* types (Figure 2). The majority of isolates belonged to *spa* type t632 and t657 (9/66; 13.6% each *spa* type). Over half of the *spa* types were represented by 1–2 isolates only. Using MLST, isolates were assigned into 14 different sequence types (ST772, ST239, ST8, ST22, ST6, ST30, ST1413, ST672, ST1, ST526, ST45, ST238, ST1482, ST207) (Figure 2 and Figure 3). Of these STs, ST772 and ST239 were the prevalent STSs that constituted 24.2% (16/66) and 22.7% (15/66) of isolates, respectively. ST1, ST45, ST526, ST238, ST1482 and ST207 were each represented by 1.5% (1/66) of isolates. Comparing the MLST to PFGE results, isolates grouped in the same PFGE cluster were assigned to the same ST. For example, all isolates in PFGE clusters A, B, D, and H were assigned to ST8, ST772, ST239, and ST1413, respectively. However, two different STs were identified in clusters C (ST239 and ST238), F (ST30 and ST1482), and G (ST22 and ST207) and three different STs in cluster E (ST6, ST672 and ST1). All MRSA isolates were distributed within six different CC types, CC8 (23; 34.8%), CC1 (17; 25.7%), CC5 (6; 9%), CC22 (6; 9.0%), CC30 (6; 9%) and CC45 (1; 1.5%) (Figure 2). CC8 was the most frequent clonal type followed by CC1. CC8 consisted of six different *spa* types including t632, one of the most prevalent *spa* types. CC8 also contained isolates belonging to sequence type ST239 (15; 22.7%), considered a pandemic strain, followed by CC1 with sixdifferent *spa* types. The most common clonal types were ST772-CC1-t657 (*n* = 9) followed by ST239-CC-8-t632 (*n* = 8), ST239-CC8-t030 (*n* = 5), ST8-CC8-t008 (*n* = 5) and ST30-CC30-t021 (*n* = 5). Clonal types of sequence type ST672, ST207 and ST1413 were unknown.

## 4. Discussion

Like other geographical regions around the globe, MRSA is a serious health concern in Pakistan [28]. MRSA emergence was reported for the first time in the 1960s in the United Kingdom and since then its prevalence has been increasing throughout the world [8]. In the present study, high prevalence of MRSA (88%) among the collected *S. aureus* was observed while only 12% isolates were MSSA. Although high prevalence of *mec*A in clinical *S. aureus* isolates has also been reported in Pakistan previously, this work is one of those reporting the highest frequency of MRSA [15,17,28]. For example, a study conducted by Siddiqui et al. on clinical isolates in Karachi reported 52% prevalence of MRSA among *S. aureus* isolates, while 68% of the isolates were MDR *S. aureus* [29]. A similar study conducted in Peshawar city of Pakistan on clinical isolates by Ullah et al. reported prevalence of 36% [28]. Furthermore, a recent study conducted at a tertiary care hospital of Islamabad city of Pakistan reported 65% prevalence of MRSA isolates; 83% of those MRSA isolates were MDR *S. aureus* [13]. However, it is important to note that MRSA prevalence data may differ from town to town and even between hospitals of the same city [16]. Therefore, additional studies will provide needed data to inform decisions in specific clinical settings and to increase positive outcomes with infections caused by MRSA. The results of this study are alarming, since the majority of the isolates were resistant to drugs commonly used to treat *S. aureus* infections. Inappropriate use of antibiotics in Pakistan is widespread as physicians prescribe the antibiotics without performing sensitivity tests [30]. Self-medication along with inappropriate dispensing of antibiotics among patients are some of the other weaknesses in the clinical health system in Pakistan that further enhance the prevalence of antibiotic resistance [31,32]. In contrast to the previous reported results of different studies [12,33,34], no vancomycin resistant *S. aureus* were identified in the present study. Vancomycin intermediate resistant and vancomycin resistant *S. aureus* (VISA and VRSA, respectively) are a serious public health concern. In Pakistan, VISA was reported for the first time in 2004 while VRSA was reported for the first time in 2009 [14,35]. Concern for emergence of VISA increased after the study conducted by Hakim et al. (2007) which reported an incidence rate of 13% of VISA isolates [33]. According to some of the studies conducted on usage of antibiotics in Pakistan, the most commonly used antibiotics are penicillin, cephalosporins, fluoroquinolones, aminoglycosides, ceftriaxone, cefradine, ciprofloxacin and tetracycline [32,36,37,38], which would account for the resistance patterns among MRSA observed in this study.

The hospital sampled in this study receives patient samples from different neighboring cities, which was reflected in the genetic diversity of the population structure of the isolated strains. In the present study, MRSA isolates exhibited 23 different *spa* types. Although a paucity of data is available on *spa* typing in Pakistan, approximately 10 *spa* types from this study have been previously reported in other studies. For example, Syed et al. (2018) reported *spa* type t657 in Pakistan from table egg samples [39] while *spa* types t632, t030, t021, t008, t5414, t314, t345, t127 and t064 detected in the present study have also been reported in Pakistan, primarily from clinical samples [13,39,40]. To the best of our knowledge, no previous record is available for Pakistan on most *spa* types identified for the first time in this study (t657, t304, t1309, t223, t852, t345, t790, t525, t331, t10234, t14125, t711, t1991 and t6100).

ST772-CC1-t657 or the Bengal Bay clone was one of the most identified clones in this study. This clone was first reported in Bangladesh and has spread to other locations due to travel to India or contact with travelers to that region [41,42]. It is known to be endemic in regions around the Bay of Bengal and resulted in the discovery of its first clinical case which was associated with surrounding regions [18,43]. This clone was first classified as HA-MRSA and caused health care issues in multiple countries as defined in other studies [12,28,41,44,45]. It is now also associated with community infections and has linked Pakistan with the Arabian Peninsula epidemiologically as described previously [18]. The diversity of MRSA clones in this study may also be due to travel or the expansion of clones within the hospital or community. Two other prevalent clones in this study, ST239-CC8-t632 and ST239-CC8-t030, have also been recently identified as dominant epidemic MRSA clones from clinical specimens from hospitals in China [46]. According to that study, ST239-t030 has been a dominant epidemic clone in China for the last 20 years, displacing the previous dominant clone, ST239-t037. ST239 was also once considered to be hospital-associated MRSA (HA-MRSA), but it is no longer confined to hospitals only and has been reported as a commonly circulating strain in Middle East and Gulf countries [17,18].

Like many of the MRSA clones detected in this study, ST30-CC30-t021 has been described in different regions of the world and from various sources. For example, it was one of four major MRSA clades detected in Argentina between 2004 and 2015 while also circulating among a veterinarian and a dog from the same veterinary clinic in Japan [47,48]. Recently, the USA300 pulsotype (ST8-CC8-t008) has been detected in studies of clinical MRSA isolates from Pakistan [17,18,49]. Some of these same clones from Pakistan show a close epidemiological association of Pakistan with Arab Gulf countries and the Middle East, as approximately 3.6 million Pakistanis are employed there [17]. The proportion of CC5 and CC22 in isolates from the present study was 9.0% each while CC30 was the fourth most abundant clonal complex found. This clonal complex was reported for the first time in the UK but has now disseminated to Australia, Belgium, Canada, Greece, Finland, Denmark, Ireland, Spain, Sweden and the US [50]. The CC30/ST30 clonal type was also previously reported in Pakistan [51]; six isolates in the present study were found to belong to this clonal type. However, other MRSA clones reported previously in studies conducted in Pakistan, such as CC509 and CC6, were not found in this study [17,18].

## 5. Conclusions

Antibiotic resistance remains a serious health concern globally as well as in local hospitals. The results of the current study present an alarming situation as frequency of MRSA was very high among the *S. aureus* isolated. The genetic fingerprinting results indicate that the MRSA population of the present study is diverse and comprised of several global and regional clones. Monitoring evolution and circulation of antimicrobial resistant bacterial clones in the clinical setting allows for: (1) making informed decisions in prescribing the most effective antimicrobial treatment therapy for patients and (2) forming strategies to develop effective control measures to limit dissemination of these clones.

## Figures and Tables

**Table 1:** Multidrug resistance patterns among clinical *Staphylococcus aureus*.

	No. Isolates		No. Antibiotic Classes
Resistance Pattern ^a^	MRSA	MSSA	
AmpCefCipCliEryGatGenLevOxaPenRifTetTri	1	0	7
AmpCefCipCliEryGatGenLevOxaPenRifTet	8	1	6
AmpCefCipEryGatGenLevOxaPenTetTri	0	1	6
AmpCefCipEryGatGenOxaPenRifTet	8	1	6
AmpCefCipEryGatLevOxaPenTet	1	0	4
AmpCefCipEryGatLevOxaPen	1	0	3
AmpCefCipGatGenLevOxaPen	2	0	3
AmpCefCipGatLevOxaPen	1	0	2
AmpCipCliEryGatGenLevOxaPen	1	0	4
AmpCipEryGatGenLevOxaPen	0	1	4
AmpCipEryGatLevOxaPen	5	0	3
AmpCipEryGatLevOxaPenTet	3	0	4
AmpCipEryGatLevPenTet	0	2	4
AmpCipGatGenLevOxaPen	6	0	3
AmpCipGatGenLevOxaPenTet	1	0	4
AmpCipGatGenLevOxaPenTri	1	0	4
AmpCipGatLevOxaPenTet	2	0	3
AmpCipGatLevOxaPen	7	0	2
AmpEryOxaPen	1	0	2
AmpOxaPen	8	0	1
AmpOxaPenTetTri	1	0	3
AmpOxaPenTet	4	0	2
AmpPenTet	2	1	2
AmpPen	1	0	1
CipGatLevOxaPen	1	0	2
CipGatLevOxa	0	1	2
Pan-susceptible	1	1	0

^a^ Amp, ampicillin; Cef, ceftriaxone; Cip, ciprofloxacin; Cli, clindamycin; Ery, erythromycin; Gat, gatifloxacin; Gen, gentamicin; Lev, levofloxacin; Oxa, oxacillin; Pen, penicillin; Rif, rifampin; Tet, tetracycline; Tri. Trimethoprim/sulfamethoxazole.

## Data Availability

Data is contained within the article.
